# Continuous-Flow Technology for Chemical Rearrangements: A Powerful Tool to Generate Pharmaceutically Relevant Compounds

**DOI:** 10.1021/acsmedchemlett.3c00010

**Published:** 2023-02-03

**Authors:** Antonella Ilenia Alfano, Sveva Pelliccia, Giacomo Rossino, Orazio Chianese, Vincenzo Summa, Simona Collina, Margherita Brindisi

**Affiliations:** ∥ Department of Pharmacy (DoE 2023-2027), University of Naples Federico II, via D. Montesano 49, 80131, Naples, Italy; ‡ Genetic S.p.A., Via Canfora, 64, 84084 Fisciano (Salerno), Italy; § Department of Drug Sciences, University of Pavia, Via Taramelli 12, 27100 Pavia, Italy

**Keywords:** Curtius rearrangement, Hofmann rearrangement, Schmidt rearrangement, flow chemistry, sustainable synthesis, safety

## Abstract

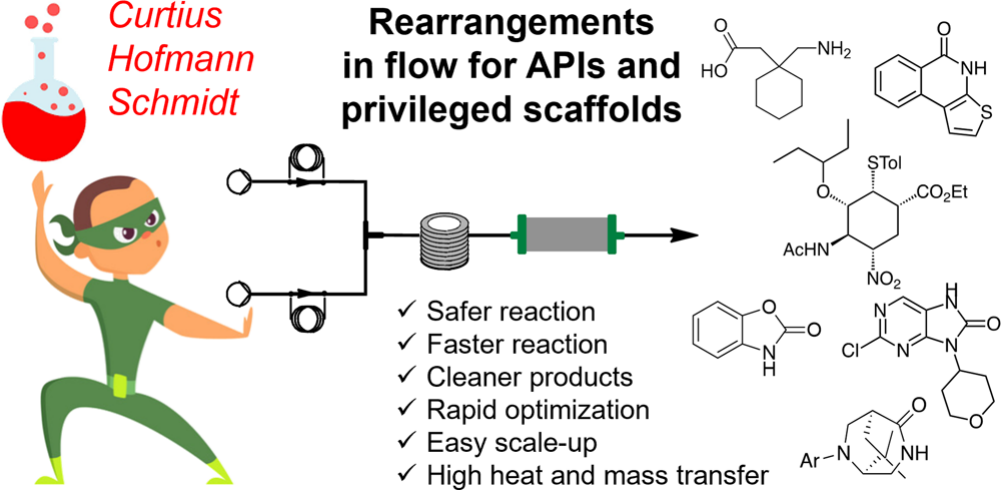

The efficacy, safety, and scale-up of several chemical rearrangements remain unsolved problems due to the associated handling of hazardous, toxic, and pollutant chemicals and high-risk intermediates. For many years batch processes have been considered the only possibility to drive these reactions, but continuous-flow technology has emerged, for both academic laboratories and pharmaceutical companies, as a powerful tool for easy, controlled, and safer chemistry protocols, helping to minimize the formation of side products and increase reaction yields. This Technology Note summarizes recently reported chemical rearrangements using continuous-flow approaches, with a focus on Curtius, Hofmann, and Schmidt reactions. Flow protocols, general advantages and safety aspects, and reaction scope for the generation of both privileged scaffolds and active pharmaceutical ingredients will be showcased.

The increasing need for safer and more sustainable practices has prompted chemists to re-evaluate and re-design several synthetic protocols and strategies. Accordingly, the pharmaceutical industry is in search of enabling technologies to reduce process footprint, minimize lead time, and accelerate scale-up.

Flow chemistry allows the development of protocols for single chemical reactions or multi-step synthesis in a continuous fashion in flow set-ups of different scales.^1^ The general continuous-flow chemistry set-up involves different elements (Figure 1).^2^

**Figure 1:**
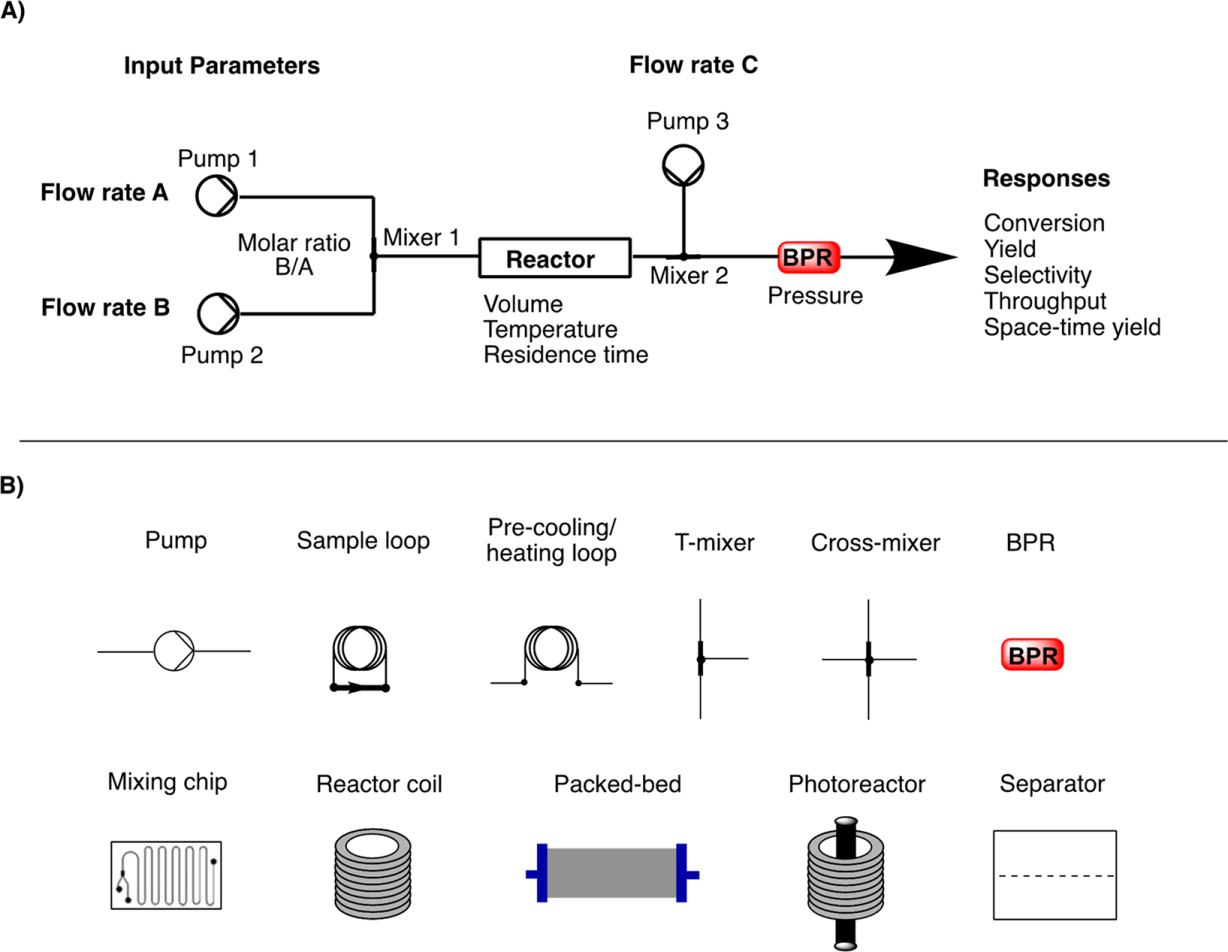
A) General representation and B) main elements of a flow chemistry set-up. BPR = back-pressure regulator.

Key advantages of flow technologies are summarized below:

i) *Faster reactions*: Flow reactors are easily pressurized, thus allowing increased heating capability, which in turn can speed-up reactions.
ii) *Cleaner products*: Flow reactors enable excellent reaction selectivity; rapid diffusion mixing avoids the issues found in batch reactors.^3^
iii) *Safer reactions*: Flow chemistry allows only a small amount of hazardous intermediate to be formed at any instant; thus a flow reaction is intrinsically safer with respect to the batch counterpart due to the lower reactor volume.^4^
iv) *Integrated work-up and analysis*: Reaction products can be flowed into an aqueous work-up system or a solid-phase scavenger column and then either be analyzed in line or, using a sampler and diluter, be injected into an LCMS.
v) *Rapid reaction optimization*: Automated flow chemistry enables the quick variation of reaction conditions even on very small scales.
vi) *Easy scale-up*: Scale-up issues are minimized due to maintaining excellent mixing and heat transfer. Three main strategies can be considered for scaling-up the flow reaction, i.e., increase the number of reactors, increase the channel length, and/or increase the channel diameter.^5^
vii) *Higher heat and mass transfer*: This is mainly due to the small dimensions, high interfacial surface of reactors, and efficient reagent mixing.^6^

Often, when conducting batch chemistry, the use of hazardous reagents and/or the generation of high-risk intermediates requires either a work-around or the devising of a new route to avoid issues of potential toxicity or extreme exothermic processes.^7,8^ This is particularly true when dealing with classical chemical rearrangements (e.g., Curtius, Hoffmann, and Schmidt rearrangements) which encompass the handling of hazardous, toxic, and/or pollutant chemicals as well as high-risk intermediates. In this context, flow chemistry can provide unique control over reaction parameters, enhancing the overall reactivity, efficiency, and safety; it can also allow to *in situ* intercept the generated intermediates, thus allowing the generation of more complex molecular architectures through multi-step transformations.

In this Technology Note we highlight recent advances in the field of flow chemistry protocols for Curtius, Hofmann, and Schmidt rearrangements applied to the synthesis of prominent functional units in several active pharmaceutical ingredients.

The Curtius rearrangement (CR) involves the thermal decomposition of an acyl azide derived from carboxylic acid to produce an isocyanate as the initial product; the latter then can easily undergo further reactions to provide amino, urea, or urethane functionalities (see Figure S1 for details).^9^ The CR has found widespread application in the synthesis of privileged scaffolds and approved drugs;^10^ however, it suffers from significant limitations related to the generation and the use of potentially explosive and highly toxic azide promoters and the corresponding acyl azide counterpart. In recent years, the application of continuous-flow protocols for CR has been explored for minimizing the use or generation of hazardous reagents, thus enabling safer, high-yielding, and environmentally friendly processes.

In 2007, Jensen et al. used the CR as a model reaction to demonstrate multi-step microchemical synthesis with in-line purification steps.^11^ Micro-separators were specifically designed to conduct liquid–liquid extraction and liquid–gas separation. Acyl azides were prepared *in situ* upon mixing of the acyl chloride with aqueous sodium azide in a microreactor, followed by a liquid/liquid micro-separator. The organic phase containing the azides was then flowed into another microreactor and heated to 90 °C to prompt CR. A gas/liquid separator was added to remove the generated nitrogen, and the final carbamates were obtained upon reaction between the generated isocyanates and the desired alcohols inserted through a third microreactor (Scheme 1A).

**Scheme 1:**
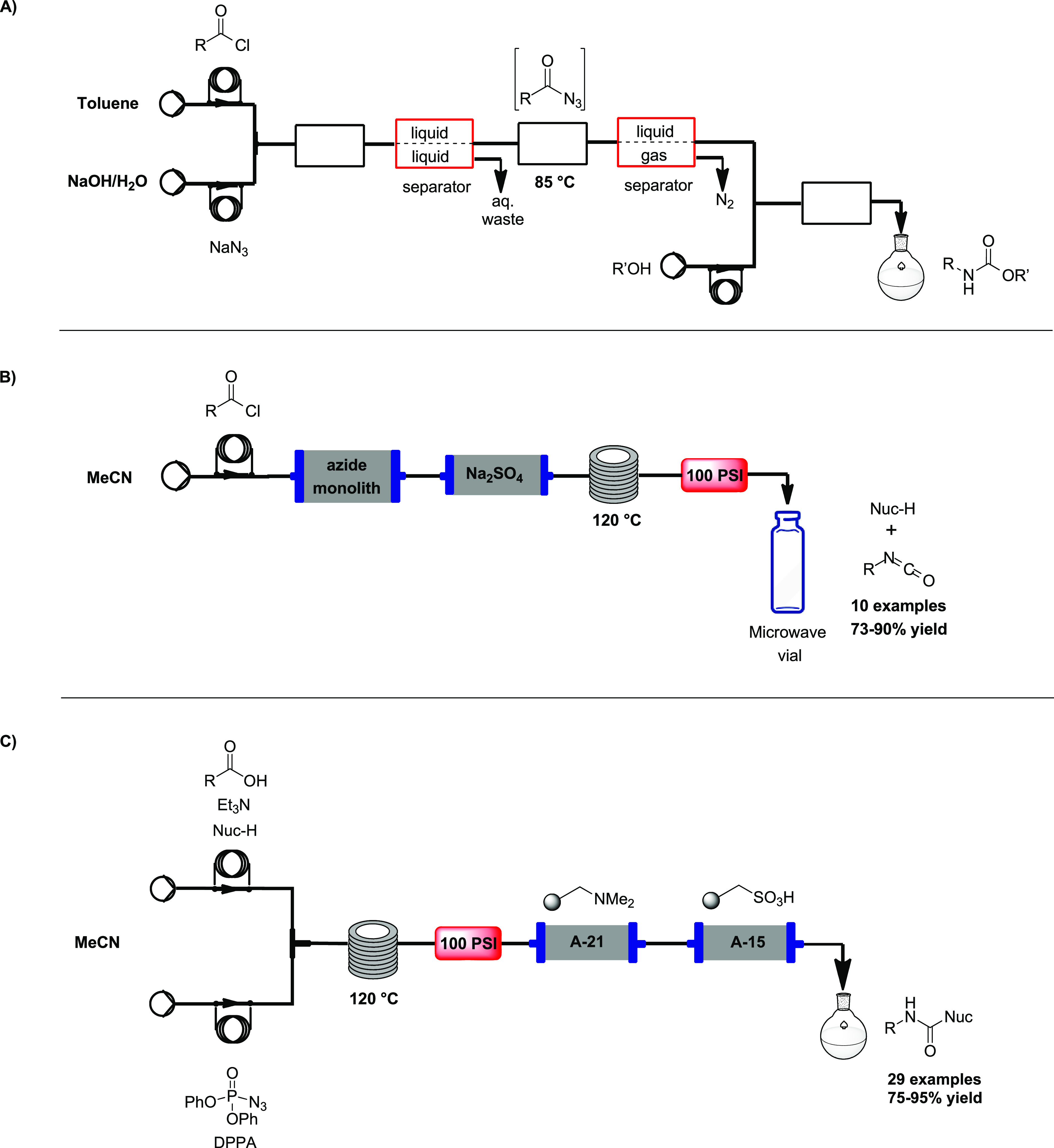
A) Flow Set-Up of the Curtius Rearrangement with In-Line Purification Steps by Jensen,^11^ B) Flow Set-Up with Azide Ion-Exchange Monolith Reactor by Baumann,^12^ and C) Curtius Rearrangement under Continuous Flow by Baumann^13^

In a follow-up paper by Baumann et al., an azide ion-exchange monolith reactor was developed for the conversion of acyl chlorides into their corresponding isocyanates via the acyl azide intermediate, to increase the safety profile of the reaction.^12^ The azide-exchange monolith was prepared directly in the flow reactor column and used in the following flow reactions. 1 mmol of a 1 M solution of the acyl chloride in MeCN was passed through a column with an azide monolith (15 mmol) for a residence time of 13 min, to guarantee complete and fast conversion. The monolith-containing column was followed by another column packed with Na_2_SO_4_ as the dehydrating agent, thus avoiding side-reactions. Subsequently, the solution was passed through a tubular flow reactor and heated to 120 °C to enable CR. The output isocyanate stream was collected in a microwave vial containing the appropriate nucleophile for the addition reaction under microwave irradiation for 10 min. A library of 10 products was generated in good yield (64–90% yields) and high purity (95%) (Scheme 1B).

In 2008, Baumann et al. developed the first fully automated CR in continuous flow, using a Vapourtec platform.^13^ A MeCN solution of triethylamine, nucleophile, and carboxylic acid was loaded into the first sample loop, while a solution of diphenyl phosphoryl azide (DPPA) in MeCN was loaded into the second one. The two streams were mixed using a simple T-piece and directly flowed into a convection-flow coil (CFC) reactor, heated to 120 °C. After residence times of 20–50 min, the flow stream passed through a column packed with a mixture of Amberlyst A-21 and A-15, to quench diphenylphosphonic acid and triethylamine. Twenty-nine compounds were obtained (73–95% yield) in >90% purity (Scheme 1C).

Hayashi and co-workers in 2011 used a flow-based CR for the synthesis of (−)-Oseltamivir phosphate (Tamiflu), a neuraminidase inhibitor for the treatment of human influenza.^14^ After the optimization of the Curtius flow protocol using TMSN_3_ as azide source, the reaction was performed in the presence of a nucleophile, to trap the generated isocyanate. A DMF solution of acyl chloride was mixed through a micromixer with a DMF solution containing TMSN_3_ and pyridine and reacted in a microreactor coil at room temperature for 20 min. The flow stream was then mixed with a solution of acetic acid and the respective nucleophile in DMF via a third stream and passed through another reactor coil at 110 °C for 70 min. A library of 19 compounds was achieved (71–100% yields, Scheme 2A). Then, the authors applied the protocol to the synthesis of Oseltamivir, starting from ethyl (1*S*,2*R*,3*S*,4*R*,5*S*)-4-(chlorocarbonyl)-5-nitro-3-(pent-3-yloxy)-2-(*p*-tolylthio)cyclohexanecarboxylate as acyl chloride and using acetic anhydride as the nucleophile. The target acetamide was achieved in 84% yield, after off-line work-up. The recrystallization of the acetamide furnished Oseltamivir, after two further reactions in batch, with high purity (Scheme 2B).

**Scheme 2:**
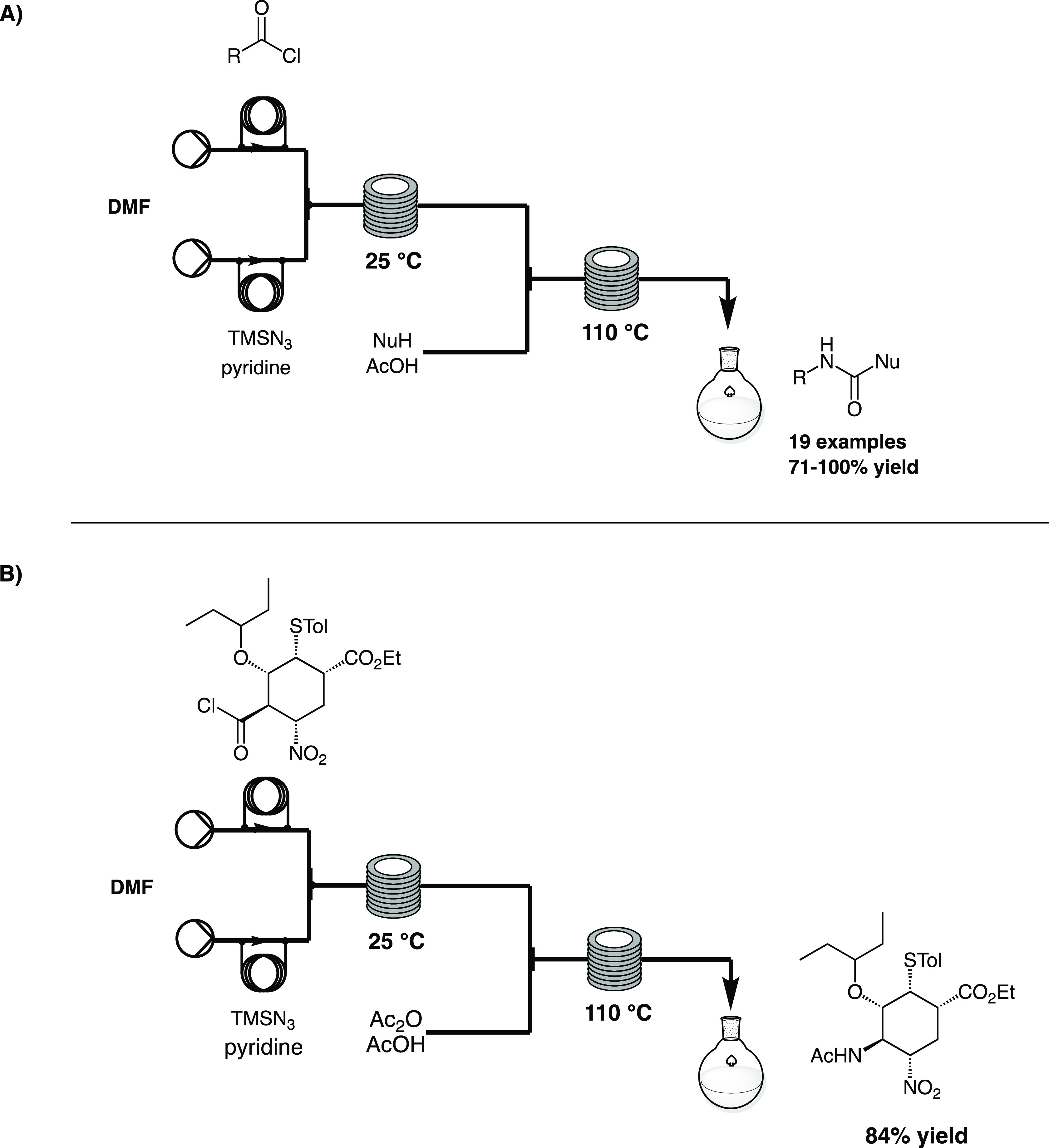
Curtius Rearrangement in Flow A) for the Synthesis of Amides and B) for the Synthesis of (−)-Oseltamivir Phosphate by Hayashi^14^

In 2014, Ley and co-workers proposed a flow protocol to synthesize bromosporine analogues as modulators of the histone reader bromodomain-containing protein 9 (BRD9).^15^ Flow chemistry helped overcome the issues associated with the use of a high quantity of DPPA and the release of a large volume of nitrogen. A solution of 3,6-dichloropyridazine-4-carboxylic acid, triethylamine, and *tert-*butanol in toluene/acetonitrile was mixed with a solution of DPPA and pumped into two different reactor coils, heated to 120 °C, to prompt CR. The use of in-line devices allowed easy monitoring of temperature and pressure to ensure process safety. After 2.3 h, the target compound was obtained with 39% yield, prone to further batch reactions (Scheme 3A).

**Scheme 3:**
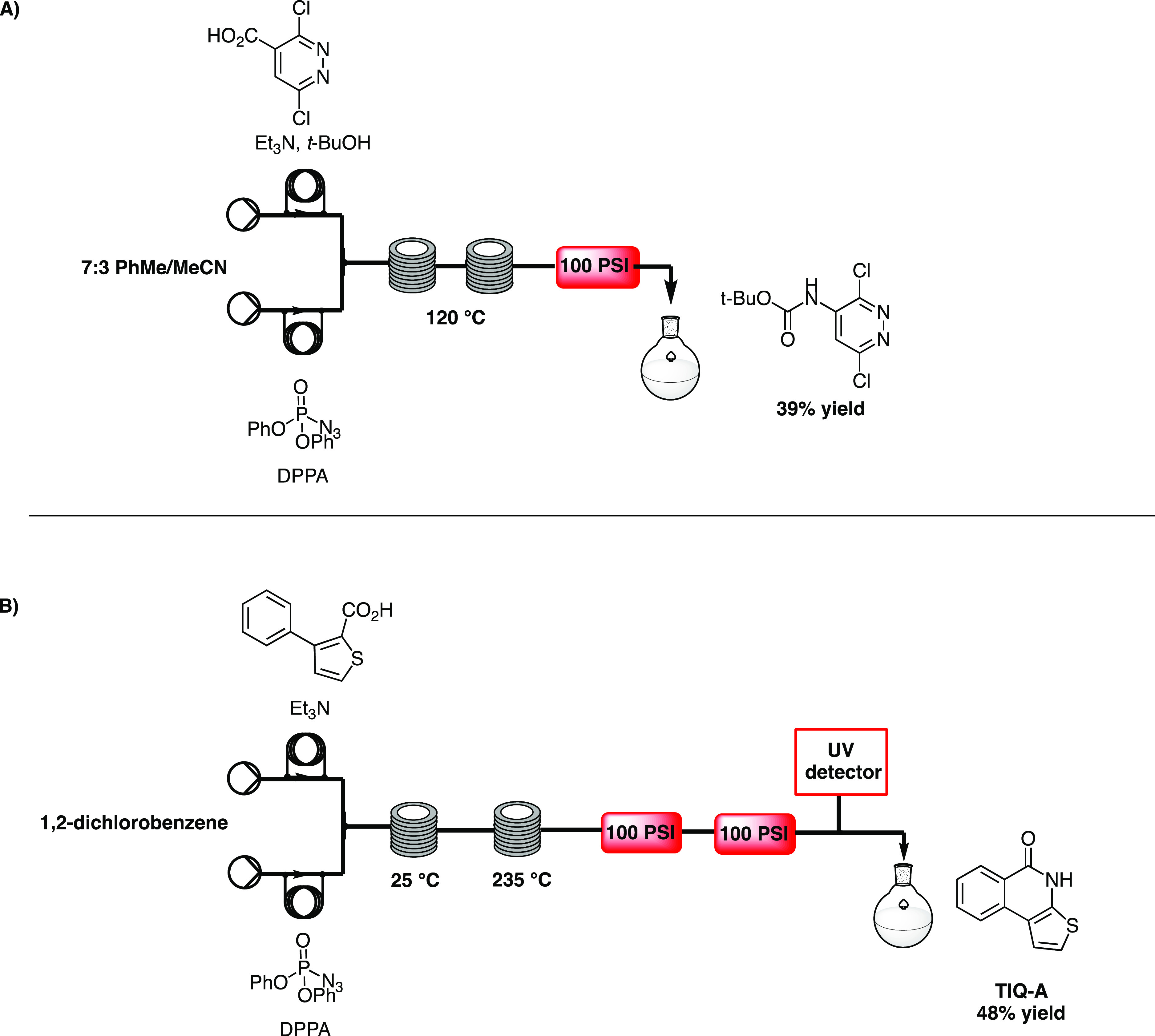
A) Flow Protocol to Synthesize Bromosporine Analogues by Ley,^15^ and B) Flow Multi-scale Synthesis of TIQ-A by Gioiello^16^

In 2014, Gioiello and co-workers^16^ reported a continuous-flow protocol for the multi-scale synthesis of thieno[2,3-*c*]isoquinolin-5(4*H*)-one-A (TIQ-A), a building block for the generation of poly [ADP-ribose] polymerase 1 (PARP-1) inhibitors, via a one-pot, two-step acyl azide synthesis and thermal cyclization. Flow chemistry could overcome the challenges associated with the batch synthesis, such as the low yield and use of hazardous chemicals. Once the Suzuki coupling continuous-flow synthesis was established for the starting material phenylthiophene-2-carboxylic acid, the corresponding acyl azide was easily prepared in flow using DPPA. A solution of acid and triethylamine in 1,2-dichlorobenzene was mixed with a solution of DPPA in 1,2-dichlorobenzene through a T-mixer and reacted in a reactor coil at 25 °C. After a residence time of 100 min, the stream passed through another coil, heated to 235 °C, to promote cyclization. TIQ-A was generated in 48% yield, compared to the 33% obtained in batch (Scheme 3B). The scale-up potential was demonstrated by synthesizing 2.7 g of TIQ-A in 50% overall yield.

In 2017, Marsini et al. developed a concise flow synthesis of a C-C chemokine receptor type 1 (CCR1) antagonist, namely 1-(4-fluorophenyl)-*N*-(1-(2-(methylsulfonyl)pyridin-4-yl)cyclopropyl)-1*H*-pyrazolo[3,4-*c*]pyridine-4-carboxamide, using a CR.^17^ Starting from a batch synthesis of 1-(2-(methylsulfonyl)pyridin-4-yl)cyclopropane-1-carboxylic acid, a first semi-continuous-flow synthesis of carbamate was developed. A solution of acid and triethylamine in toluene was mixed in a continuously stirred tank reactor (CSTR) with DPPA for a residence time of 10 min at 55 °C. The stream containing the acyl azide was collected in a batch reactor with a solution of toluene/*tert*-amyl alcohol at 100 °C to trigger CR and subsequent carbamate formation. *tert*-Pentyl (1-(2-(methylsulfonyl)pyridin-4-yl)cyclopropyl)carbamate was obtained on an 8 kg scale with 80% yield and purity >99%. Based on these results, a semi-continuous CR-acid–isocyanate coupling was developed. A solution of acid and triethylamine in toluene was mixed with a second solution containing DPPA in toluene in a jacketed static mixer and pumped in a tube-in-tube reactor (in order to remove the N_2_ gas) at 135 °C for a residence time of 3 min. The tube-in-tube was then followed by an in-line FTIR to control the complete conversion of acyl azide. The stream was then collected in a tank containing 1-(4-fluorophenyl)-1*H*-pyrazolo[3,4-*c*]pyridine-4-carboxylic acid and triethylamine at 100 °C for 1 h. The product was obtained in 76% yield after off-line work-up (Scheme 4A).

**Scheme 4:**
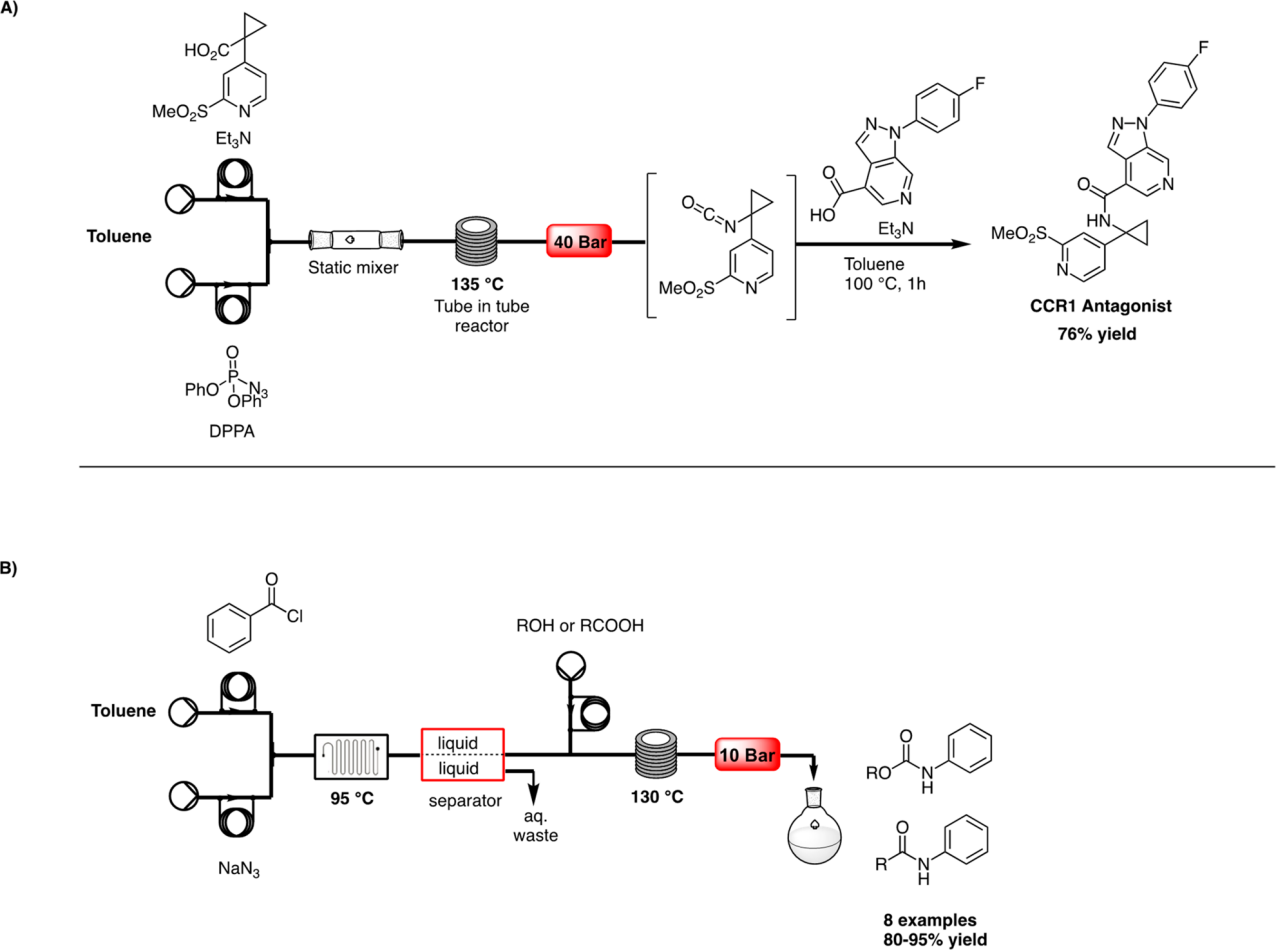
A) Continuous-Flow Synthesis of a CCR1 Antagonist by Marsini,^17^ and B) Multi-step Microreactor for Carbamates Synthesis by Sagandira^18^

In 2017, a continuous multi-step synthesis of amines, amides, and carbamates via CR was developed by Sagandira and Watts, starting from benzoyl chloride.^18^ After optimization of solvent, stoichiometry, temperature, and set-up, the best conditions were achieved for the first step. In 11.7 s, benzoyl azide was synthesized starting from 0.1 M benzoyl chloride in MeCN and 0.11 M sodium azide in water in the tube reactor. The tube was followed by a glass reactor downstream for the CR. Unfortunately, these reaction conditions did not lead to the generation of phenyl isocyanate, since the presence of water in the tubes led to the formation of the corresponding aniline. Thus, a liquid–liquid separator was added in-line after the formation of benzoyl azide, and solvent was switched from acetonitrile to toluene. A library of 7 acyl azides was obtained at 95 °C and 7.69 min of residence time (34–96% yields). A multi-step synthesis was then carried out, adding via a third stream an alcohol, or a carboxylic acid in toluene, thus obtaining a library of 9 carbamate and amide products (80–95% yields, Scheme 4B).

In 2020, Burkart et al. reported the preparation of mono- and diisocyanates in flow from renewable carboxylic acids.^19^ Starting from azelaic acid, derived from algae-sourced palmitoleic acid via ozonolysis, a bio-based isocyanate was obtained. The first and second steps, which involved a Fischer esterification and an *in situ* reaction with easily handled hydrazine hydrate, were performed in batch, due to the drawbacks associated with precipitation and subsequent clogging of the reactor. The batch intermediate was mixed with nitrous acid (which immediately generates nitrosonium ion) through a T-mixer in flow and then reacted for 97 s at 0 °C. Toluene was then added via a third stream, and the organic phase was recovered using a separator. The final product was obtained with 80% yield at 500 mg g^–1^, after passing through a dry column packed with sodium sulfate and a reactor coil heated to 85 °C in order to finalize CR. A scale-up to 100 g of heptamethylene diisocyanate furnished the product with 90% yield at 8.5 g h^–1^. This protocol was also applied to the preparation of alkyl and aromatic isocyanates, and a library of 16 products was achieved (50–90% yields, Scheme 5).

**Scheme 5:**
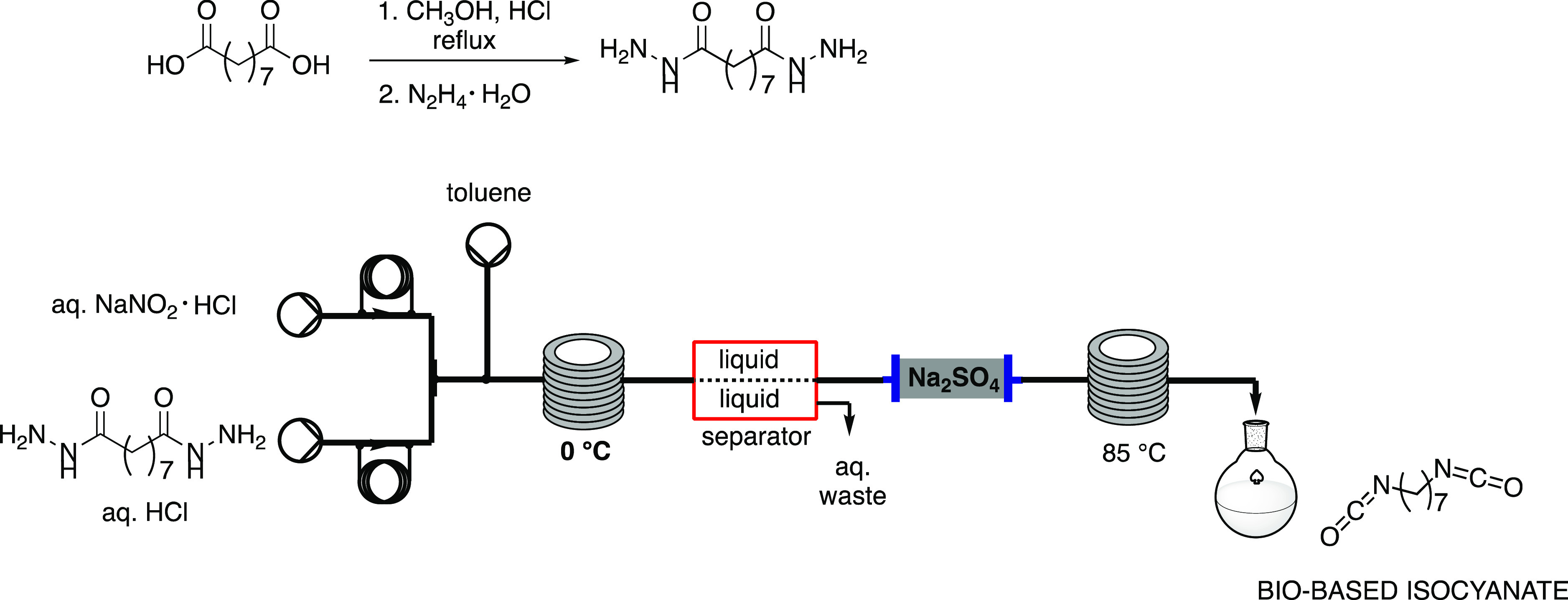
Continuous-Flow Synthesis of Bio-Based Isocyanates by Burkart^19^

In 2021, Baumann and co-workers developed a merging protocol between CR and a biocatalytic impurity tagging strategy.^20^ A solution containing different carboxylic acids, an excess of benzyl alcohol (1.8 equiv), and triethylamine in toluene was loaded in the first sample loop; the second loop was filled with DPPA in toluene. The reaction takes place in a reactor coil heated to 120 °C, followed by a 100 psi BPR to control the pressure after the release of nitrogen. The BPR was then followed by a column packed with a mixture of Amberlysts A-21 and A-15 in order to remove triethylamine, the excess of acid, and the generated diphenylphosphonic acid. Thus, the exiting stream contained only the desired product and the unreacted benzyl alcohol. Due to the harsh conditions needed to remove benzyl alcohol, a biocatalytic approach was envisaged, by adding a second column packed with *Candida antarctica* lipase B (CALB), right after a third stream containing vinyl butyrate. CALB converts benzyl alcohol in the presence of vinyl butyrate into benzyl butyrate and acetaldehyde, thus simplifying the final purification stages. A library of 13 Cbz-carbamate products was generated (48–100% yields, Scheme 6A).

**Scheme 6:**
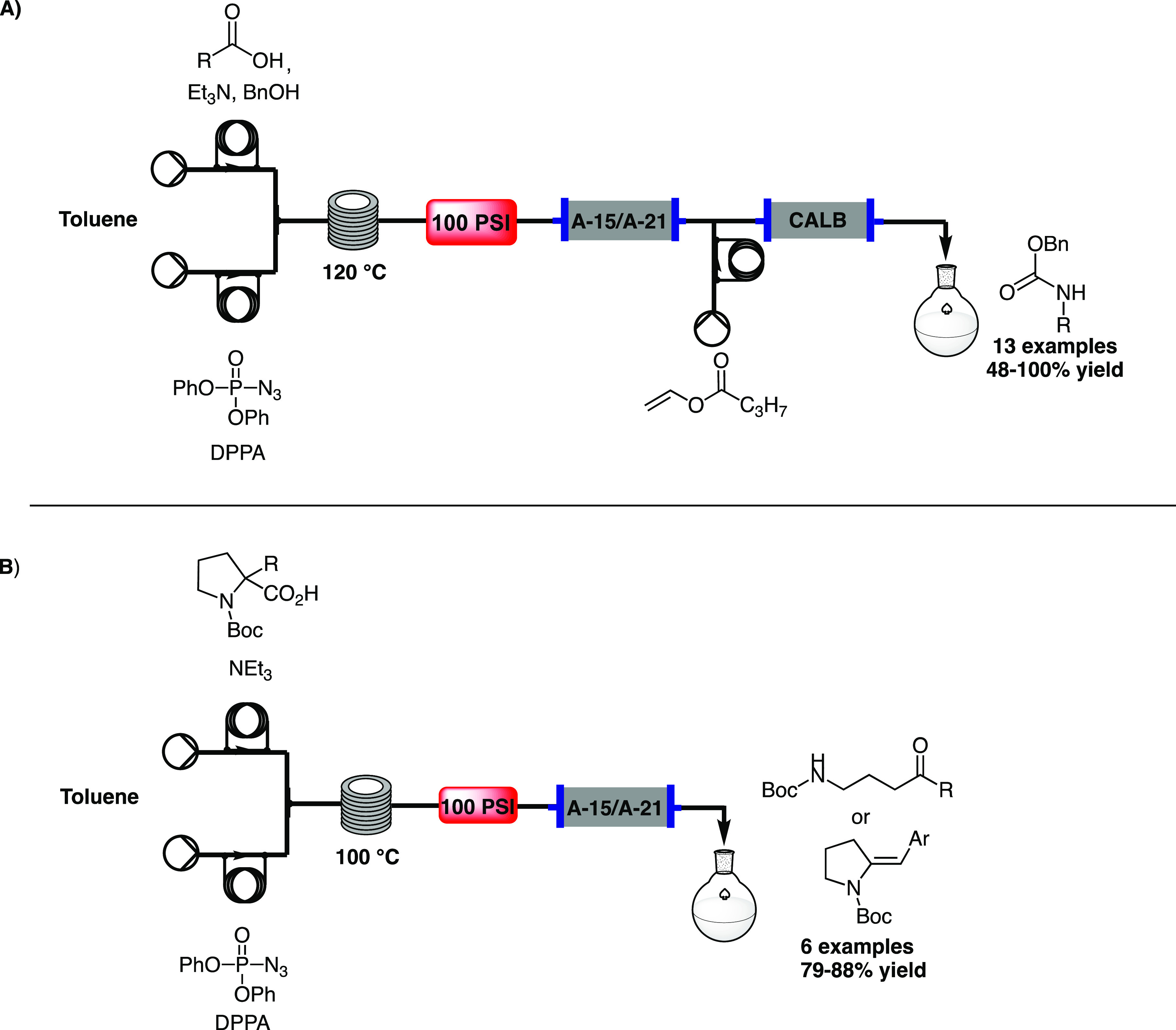
A) Flow Protocol for CR and Biocatalytic Impurity Tagging by Baumann,^20^ and B) Flow Route for Quaternary *N*-Boc Proline Moiety by Baumann^21^

In 2021, a flow route for a quaternary *N*-Boc proline moiety under thermal CR was reported by Baumann et al.^21^ A solution of the *N*-Boc proline derivative and triethylamine in toluene was mixed with a solution of DPPA in toluene and flowed into a reactor coil heated to 100 °C for 20 min. The reactor coil was followed by a 100 psi BPR and a scavenger column packed with Amberlysts A-21 and A-15. Based on the *N*-Boc proline substitution pattern, two different pathways were observed in the reaction: in the case of small aliphatic substituents, a fragmentation of the isocyanate with a subsequent ring-opening to form γ-amino ketone products was predominant; on the other side, the presence of benzylic groups gave a tautomerization of the *N*-acyliminium intermediate to provide unsaturated pyrrolidines. A library of 3 acyclic products and 3 unsaturated pyrrolidines was achieved (79–88% yield, Scheme 6B).

In 2022, Browne and co-workers^22^ reported a flow protocol for the generation of acylketene precursors. After the development of a library, the authors also demonstrated the generation and combination of two intermediates via nitrogen extrusion (Scheme 7A), synthesizing an isocyanate *in situ* through CR. Starting from a toluene solution of diazodimedone and phenylacyl azide as precursor for the generation of isocyanate, the subsequent cycloaddition reaction of the acylketene with isocyanates provided the target 1,3-oxazine-2,4-dione in 64% yield, after a residence time of 40 min in a reactor coil heated to 130 °C.

**Scheme 7:**
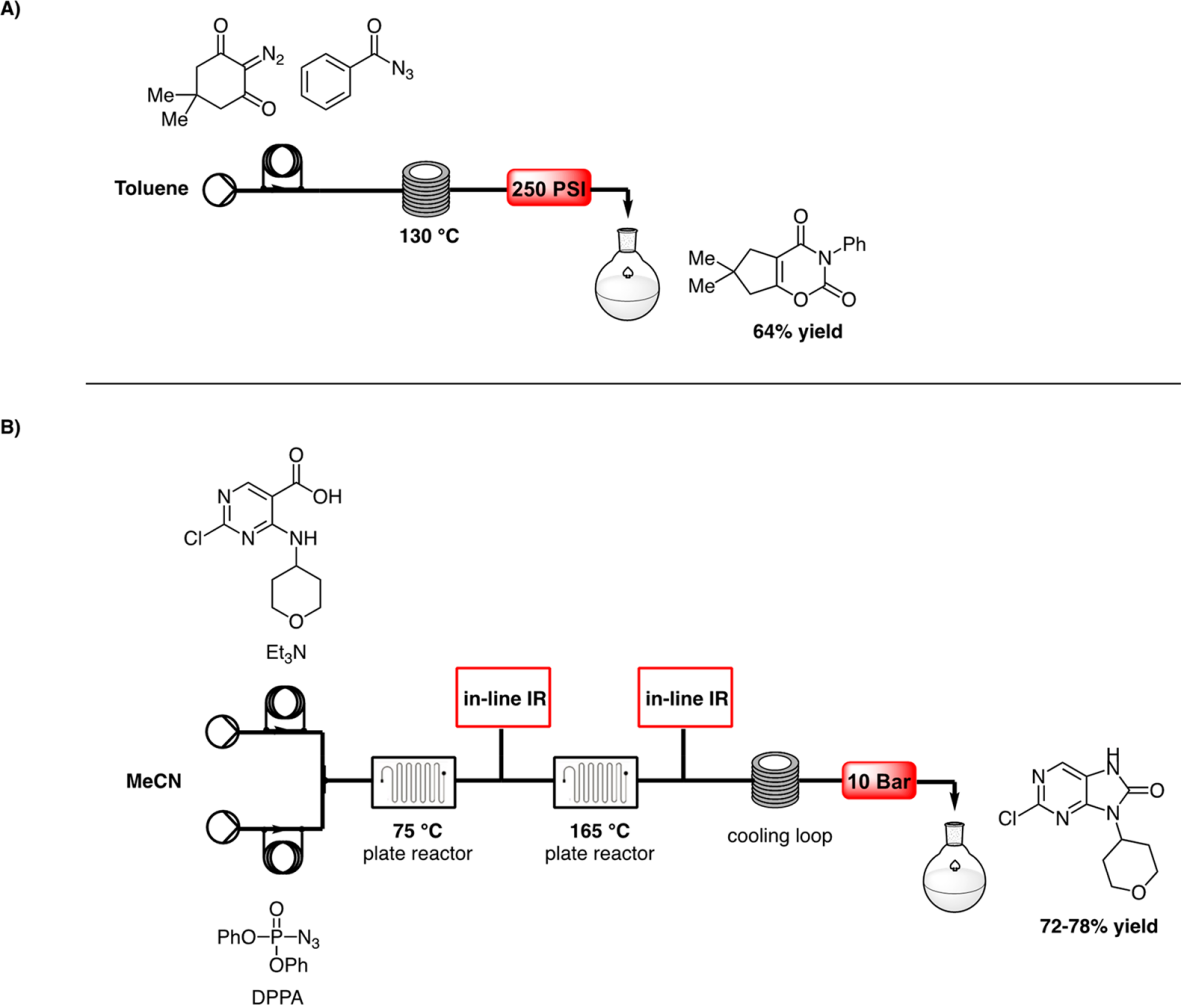
A) Flow Protocol for the Generation of Acylketene Precursors by Browne,^22^ and B) Curtius Rearrangement Using a Continuous-Flow Protocol by Mallia^23^

The most recent work at the time this review was compiled comes from Mallia et al.,^23^ who developed a flow-based CR for large-scale synthesis of AZD7648, an anticancer agent acting as a selective inhibitor of DNA-dependent protein kinase. Flow chemistry could overcome issues related to scale-up of batch synthesis, including the high quantity of explosive intermediates and the increased levels of impurities. The Cl-purinone intermediate was synthesized using a FlowStart Evo continuous set-up to optimize the parameters and find the best conditions. A solution of carboxylic acid and base in acetonitrile was mixed with DPPA to generate the acyl azide in a reactor coil at 75 °C. Rearrangement of acyl azide to isocyanate and the subsequent intramolecular cyclization take place in a second reactor heated to 165 °C. Notably, the real-time monitoring of both reactions with an in-line IR detector allowed reaction monitoring while also controlling impurity generation. An increase in yield to 72–78% was observed with respect to 61% of the batch counterpart (Scheme 7B).

The Hofmann rearrangement (HR) is a well-recognized method to convert primary carboxamides to amines or carbamates, characterized by the reduction of one carbon unit (see Figure S2 for details).^24^ The toxicity of bromine (or bromine equivalent) and the high reaction temperatures strongly limit its use in conventional laboratory settings. Flow chemistry thus represents an appealing option for the implementation of efficient and safer HR protocols, and also for drug discovery purposes.

In 2009, Palmieri et al. reported the optimization of continuous-flow synthesis of aromatic carbamates starting from aromatic amides, using the Advion NanoTek LF microreactor platform.^25^ Two different flow streams were combined in a T-mixing piece before entering in a microreactor coil heated to 120 °C. In the first stream, the selected amide was mixed with 1,8-diazabicyclo[5.4.0]undec-7-ene (DBU) as the base and an alcohol (methanol or ethanol) in order to trap the formed isocyanate, while the second stream contained the brominating agent in methanol or ethanol. A residence time of only 1 min was required for reaction completion; thus, a library of 14 compounds was easily generated (32–80% yield), displaying >97% purity (Scheme 8A). Moreover, reaction parameters were easily transferred to the Uniqsis FlowSyn continuous-flow reactor for efficient scaling-up on a gram scale.

**Scheme 8:**
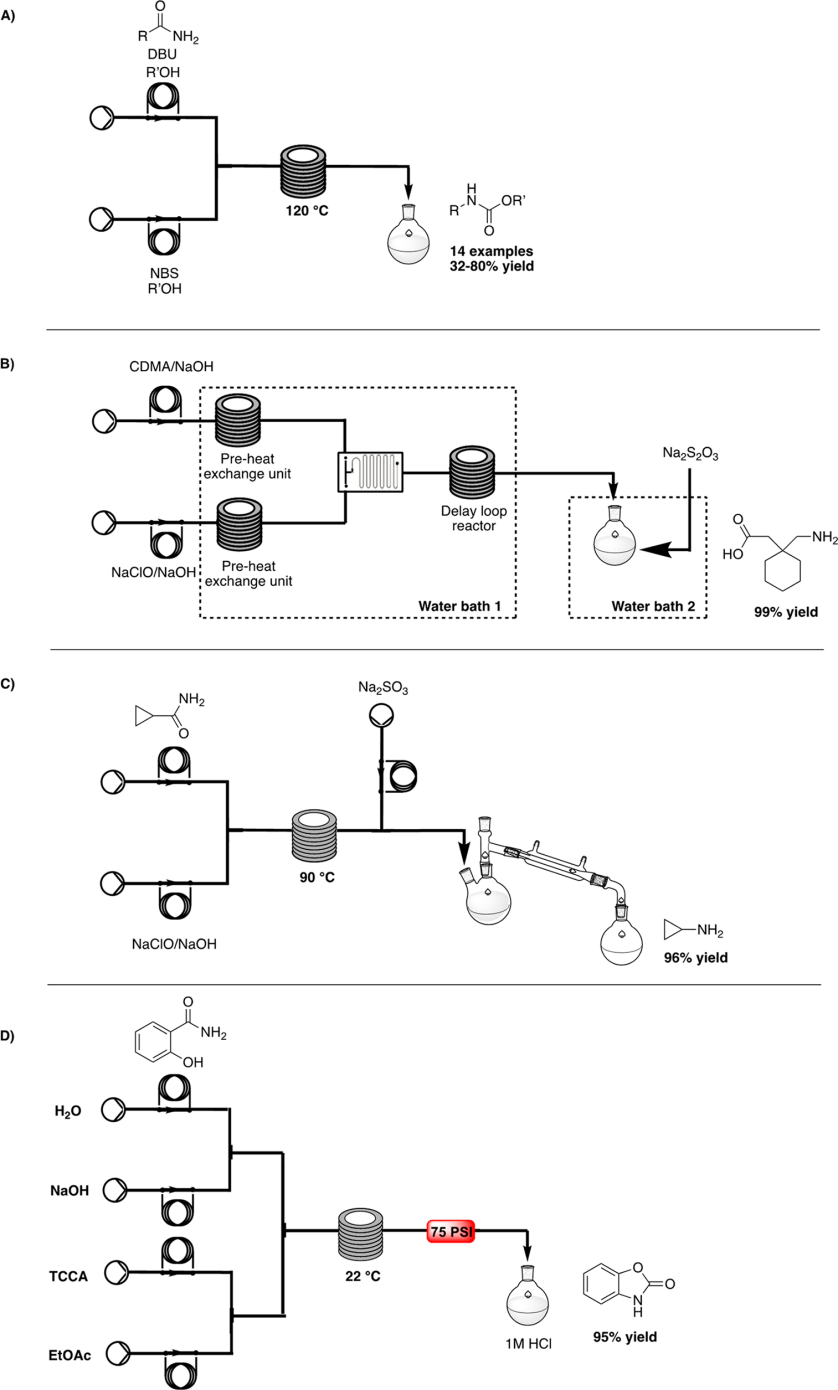
A) Continuous-Flow Synthesis of Aromatic Carbamates by Palmieri,^25^ B) Continuous-Flow Synthesis for Gabapentin by Xu,^26^ C) Continuous-Flow Synthesis of Cyclopropylamine by Xu,^28^ and D) TCCA-Based Chlorination under Flow Conditions by Baxendale^29^

In 2017, Xu and co-workers proposed a continuous protocol for Gabapentin, an anticonvulsant drug,^26^ to overcome the issues of a batch reactor associated with temperature and mixing control. A pre-heated solution of 1,1-cyclohexanediacetic acid monoamide (CAM) in sodium hydroxide was mixed with a pre-heated solution of sodium hypochlorite in sodium hydroxide in a micropore dispersion reactor, followed by a delay loop reactor. The micromixer and the delay loop reactor were immersed in a water bath heated to 40 °C. The best residence time was identified to be 5–7 min, and the stream was collected in a termination unit containing sodium thiosulfate to stop the reaction and quench the excess sodium hypochlorite. The Gabapentin was finally obtained through acidification and crystallization processes in >99% yield (Scheme 8B).

An efficient continuous-flow synthesis of cyclopropylamine, a chemical intermediate for floxacin antibiotics^27^ and anti-HIV drug, was developed in 2019 by Xu et al.^28^ The reported batch procedure for cyclopropylamine synthesis requires the use of cyclopropanecarboxamide and NaClO, forming the *N*-chlorocyclopropanecarboxamide, which is subsequently converted into isocyanate and hydrolyzed with sodium hydroxide. Despite the high yield (around 90%), this method is associated with side reactions, long reaction times, and safety issues. In the proposed continuous-flow protocol, a solution of cyclopropanecarboxamide at room temperature was mixed through a T-shaped micromixer with a solution of sodium hydroxide in sodium hypochlorite, with a 4 min residence time at 90 °C, a subsequent quench reaction using sodium sulfite, and distillation of the final compound. This procedure provided cyclopropylamine in 96% yield and avoided byproduct formation (Scheme 8C).

The preparation of 2-benzoxazolinone, a privileged motif in anticancer, antimycobacterial, anti-HIV, anticonvulsant, and anxiolytic agents, was recently reported by Baxendale and co-workers through an unprecedented trichloroisocyanuric acid (TCCA)-based chlorination under flow conditions via HR, starting from salicylamide.^29^ First, a two-pump system was used: the first stream, containing a 0.5 M amide in a 1 M aqueous solution of NaOH, was combined with a 0.165 M solution of TCCA in ethyl acetate and reacted for a residence time of 5 min in a reactor coil at 22–25 °C. This biphasic solution was then quenched into a 1 M solution of HCl for 40 min, giving an 18.5% yield of 2-benzoxazolinone. Due to the low yield, the high presence of different chlorinated side-products, and the low throughput, especially when running larger scale reactions, a new flow set-up, involving the use of four different pumps, was developed. A 1.5 M aqueous solution of amide, a 2.5 M solution of NaOH, a 0.3 M solution of TCCA in EtOAc, and AcOEt were mixed and reacted in a reactor coil at 22 °C for a residence time of 30 s and then quenched with a 2 M solution of hydrochloric acid. The biphasic solution was collected into a separatory funnel, and the organic phase was recovered. The target compound was achieved on a large scale (188 g) with an isolated yield of 95% and space-time yield of 1.56 g h^–1^ mL^–1^ (Scheme 8D).

The Schmidt rearrangement (SR) involves a carbonyl derivative (aldehyde, ketone, or carboxylic acid) which, under acidic conditions, provides the corresponding amine or amide derivatives, with the loss of nitrogen (see Figure S3 for details).^30^

In 2014, Jia and co-workers^31^ reported a fast and safe SR, carried out in a continuous-flow microreactor to form amides. In this case, the problems associated with the cryogenic operations and large azide consumption could be overcome through the use of flow chemistry. A first solution, containing 85% of MsOH in DME, was mixed with the second one, containing ketone and tetrabutylammonium azide (TBAA), an azide reagent soluble in organic solvents. The T-mixer was followed by a microreactor immersed in an oil bath and heated at 80 °C. After 5 min of residence time, a library of 18 amides was obtained (38–92% yield, Scheme 9A).

**Scheme 9:**
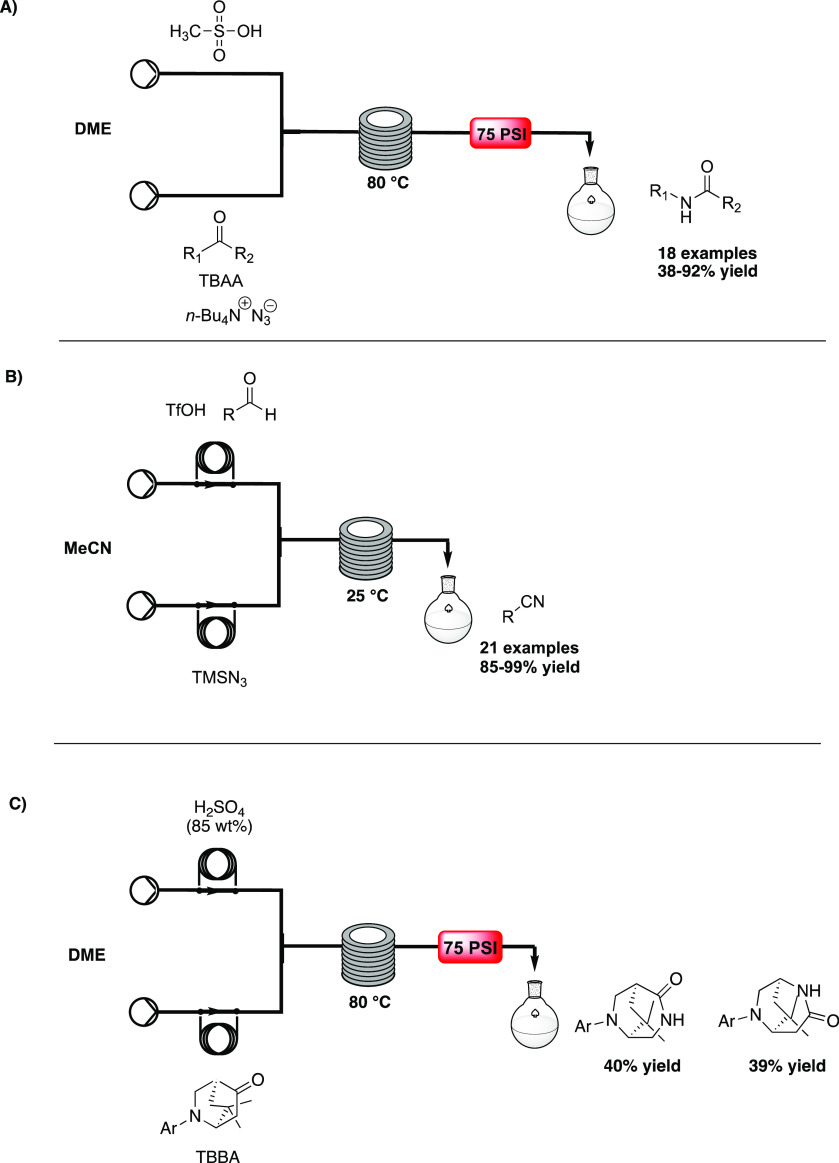
A) Continuous-Flow Schmidt Rearrangement of Ketones by Jia,^31^ B) Continuous-Flow Synthesis of Nitriles by Li,^32^ and C) Continuous-Flow Synthesis of Chiral Bicylic Homopiperazines by Brown^34^

In 2019, a continuous-flow synthesis of nitriles from aldehydes using SR was described by Li and co-workers.^32^ Nitriles represent versatile intermediates but also key pharmacophores in bioactive compounds.^33^ Multiple procedures described for SR in batch require an excess of toxic azide compounds and TsOH for the conversion, while this new continuous-flow procedure improves the safety and the scaling-up. A solution of aldehyde and TsOH acid in MeCN was mixed with a solution of TMSN_3_ in MeCN and pumped in a reactor coil at room temperature for 5 s, at 10 bar. The stream was then collected in a flask containing saturated aqueous NaClO. With these optimized conditions in hands, the authors explored the scope of the reaction, synthesizing 21 compounds (85–99% yield, Scheme 9B).

In 2021, Brown et al. reported a flow-based scale-up of chiral bicyclic homopiperazines through the SR.^34^ Batch issues related to a long reaction time and acid neutralization prompted the authors to explore continuous flow, exploiting the previous work by Jia and co-workers on the generation of the homopiperazine moiety. A solution of sulfuric acid in 1,2-dimethoxyethane (DME) was mixed with (1*R*,4*S*)-2-(4-ethoxyphenyl)-7,7-dimethyl-2-azabicyclo[2.2.2]octan-5-one^35^ and tetrabutylammonium azide in DME. The stream passed into a reactor coil heated to 80 °C, and after steady-state conditions (17 min) were achieved, the stream was collected for 3.5 h. Two different stereoisomers were obtained (1:1 ratio, yields of 39% and 40%) (Scheme 9C).

The matchless role of chemical rearrangements in the construction of more complex structures through the cleavage and reconstruction of chemical bonds justifies the search for safe, easy to scale-up, and—preferably—more sustainable protocols. In this context, flow chemistry is revolutionizing chemistry laboratories and our scientific discovery infrastructure. An increasing number of reports describe chemical rearrangements successfully achieved by means of continuous-flow operations, with the in-line generation and/or consumption of potentially hazardous reactants and intermediates, control of reaction parameters, and easy scale-up of final compounds through multiple reactors. We provided an overview on the application of flow technologies for the most common chemical rearrangements, highlighting advantages and potentialities. Massive opportunities remain for pioneering science and engineering from this expanding field in drug discovery. However, a well-trained workforce is still needed for the industrial continuous-flow production of active pharmaceutical ingredients.

## Supporting Information Available

Rearrangement mechanisms and description (PDF)

## Abbreviations

BPR: back-pressure regulator
BRD9: bromodomain-containing protein 9
CALB: *Candida antarctica* lipase B
CAM: 1,1-cyclohexanediacetic acid monoamide
CCR1: C–C chemokine receptor type 1
CFC: convection-flow coil
CR: Curtius rearrangement
CSTR: continuously stirred tank reactor
DBU: 1,8-diazabicyclo[5.4.0]undec-7-ene
DME: 1,2-dimethoxyethane
DMF: dimethylformamide
DPPA: diphenyl phosphoryl azide
HR: Hofmann rearrangement
PARP-1: poly[ADP-ribose] polymerase 1
SR: Schmidt rearrangement
TBAA: tetrabutylammonium azide
TCCA: trichloroisocyanuric acid
TIQ-A: thieno[2,3-*c*]isoquinolin-5(4*H*)-one-A
