# Supplementary material for: Continuous-Flow Technology for Chemical Rearrangements: A Powerful Tool to Generate Pharmaceutically Relevant Compounds
Source: ACS Med Chem Lett. 2023 Feb 3;14(3):326–37. doi: 10.1021/acsmedchemlett.3c00010 (PMC10009796; doi:10.1021/acsmedchemlett.3c00010)
Supplement: Supplementary file 1 — ml3c00010_si_001.pdf [file ml3c00010_si_001.pdf]

Supplementary Material

Continuous flow technology for chemical rearrangements: a powerful tool to generate pharmaceutically relevant compounds

Antonella Ilenia Alfano,[a,$] *Sveva Pelliccia,*[a,$] *Giacomo Rossino,*[b] *Orazio Chianese,*[c] *Vincenzo Summa,*[a] *Simona Collina,*[b,*] *and Margherita Brindisi*[a,*]

[a] Department of Pharmacy (DoE 2023-2027), University of Naples Federico II, via D. Montesano 49, 80131, Naples, Italy

[b] Genetic S.p.A., Via Canfora, 64, 84084 Fisciano (Salerno), Italy

[c] Department of Drug Sciences, University of Pavia, Via Taramelli 12, 27100 Pavia, Italy

[$] These authors contributed equally

Table of Contents

Curtius Rearrangement……………………………………………………..…….page S2

Hofmann Rearrangement…………………………………………………………page S3

Schmidt Rearrangement………………………………………………………….page S4

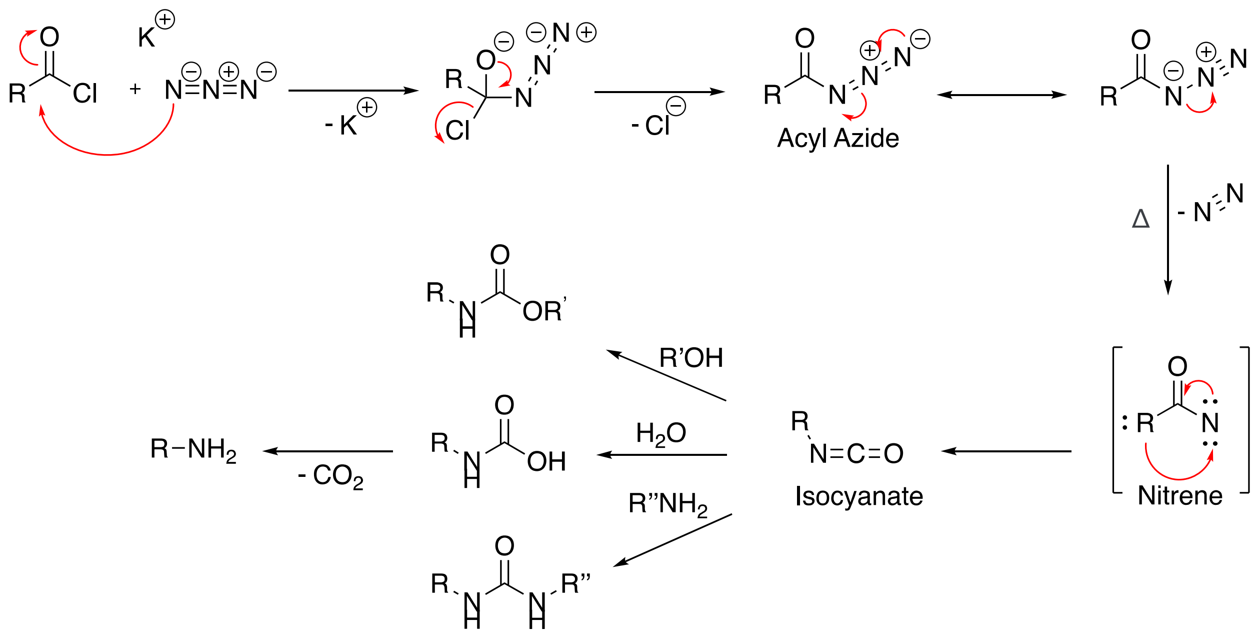

Figure S1. Mechanism of Curtius rearrangement.

The accepted mechanism of the Curtius rearrangement involves an acyl azide, deriving from carboxylic acids or acyl chlorides, which then undergoes thermal conversion into the corresponding isocyanate derivative through an acyl nitrene intermediate, with the release of N_2 (Figure S1). The isocyanate undergoes attack by a variety of nucleophiles such as water, alcohols and amines, to yield a primary amine, carbamate or urea derivative respectively. During the process, molecular nitrogen is eliminated and simultaneously a [1,2]-shift of the nucleophile attack to the carbonyl group takes place with retention of configuration. The resulting amine has a carbon less, because the last step of the reaction entails the loss of CO_2.

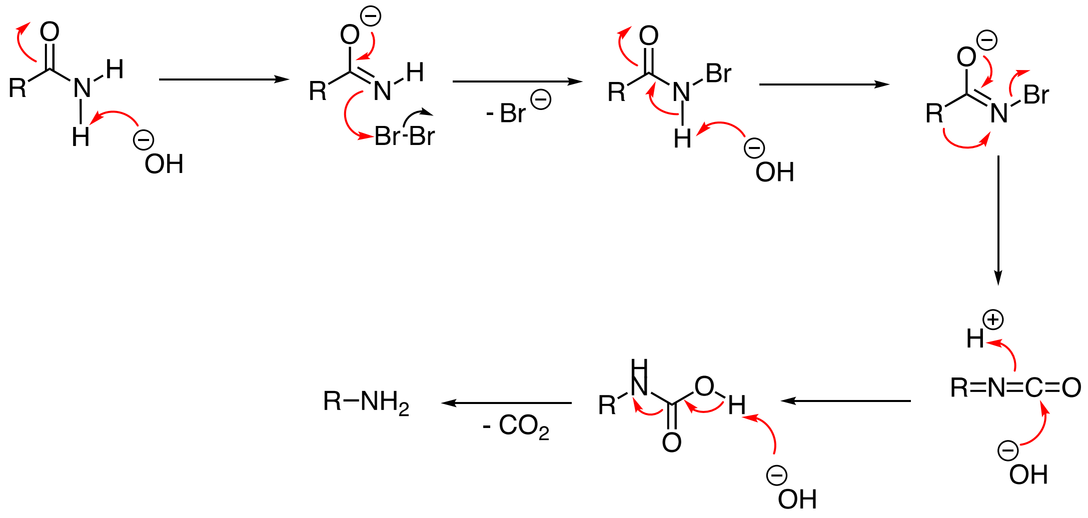

Figure S2. Mechanism of the Hofmann rearrangement

This process involves oxidation of the nitrogen followed by rearrangement of the carbonyl and nitrogen to give an isocyanate intermediate. The reaction has been adopted widely as a methodology to access the more reactive electrophilic isocyanate species that are capable of reacting with numerous nucleophiles to form amines, carbamates, and ureas. The accepted mechanism involves the treatment of an amide with bromine and a base (usually NaOH or KOH). In the first step, the base abstracts an acidic proton from the amide, forming an oxygen anion. Then, the latter reacts with bromine in an α-substitution reaction to furnish an *N*-bromoamide. A second abstraction of acidic proton, gives a bromoamide anion, that rearranges to achieve the isocyanate intermediate. This intermediate adds water in a nucleophilic addition step to provide a carbamic acid, that spontaneously loses CO_2, yielding the amine product (Figure S2).

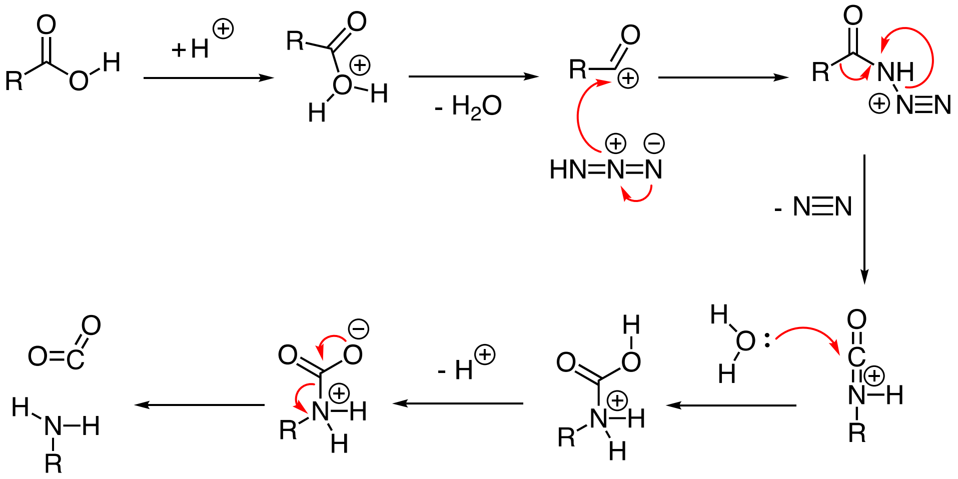

Figure S3. Mechanism of Schmidt rearrangement.

The reaction starts with the generation of an acylium ion obtained after protonation and loss of water that reacts with hydrazoic acid, forming the protonated azido ketone. The migration of the alkyl R group through a rearrangement reaction over the C-N bond with expulsion of nitrogen gives the protonated isocyanate. The nucleophilic addition of water forms the carbamate, which after deprotonation and loss of carbon dioxide provides the corresponding amine derivative (Figure S3).
